# A Receiver Perspective on Knowledge Sharing Impact on Consumer–Brand Relationship in Virtual Communities

**DOI:** 10.3389/fpsyg.2021.685959

**Published:** 2021-10-11

**Authors:** Jiemei Zhang, Shaojing Qi, Bei Lyu

**Affiliations:** ^1^Business School, Henan University, Kaifeng, China; ^2^Business School, Zhengzhou University of Economics and Business, Zhengzhou, China; ^3^School of Economics and Management, Huaibei Normal University, Huaibei, China; ^4^Chinese Graduate School, Panyapiwat Institute of Management, Nonthaburi, Thailand

**Keywords:** knowledge-sharing, sense of virtual community, product involvement, consumer-brand relationship, virtual community

## Abstract

The virtual community offers groups of consumers a knowledge-shared platform, wherein shared brand information influences the brand cognition of others. Using stimulus–organism–response (SOR) model, this study constructs a research framework for the influence of knowledge sharing on consumer–brand relationship in virtual communities. The empirical analysis shows that knowledge-sharing quality has significant positive effects on the sense of virtual community, as does the sense of virtual community on the consumer–brand relationships. This study supports the strengthening of consumer–brand relationships and the enrichment of following research on knowledge sharing of the consumer in virtual communities. Thus, companies should encourage consumers to actively participate in brand activities and focus on the development of consumer–brand relationships during participation in brand promotion.

## Introduction

Since the late 1990s, the phenomenal emergence of e-commerce has revolutionized the shopping experience, providing consumers with unprecedented convenience in online shopping. The Report of China's Internet Development, issued by CNNI in August 2019, shows that Chinese online shopping and payment accounted for 74.8% of e-commerce sales in China. With the increasing popularity of online shopping, online reviews have a growing impact on consumer purchasing decisions (Sun et al., 2020). The complexity of the social environment, the diversity of participants, the precision of intelligent information push, and the wide range of information access channels, etc. lead to the gradual decline of consumer–brand loyalty and making brand management more difficult. At the same time, consumer behavior and interaction may exist in real life, may also exist in the virtual world of small groups, consumer participation in brand information production, and consumption and dissemination as the norm. Therefore, the establishment of high-quality consumer-brand relationships, strengthen brand awareness, is an important way to improve the competitiveness of enterprises (Fu, 2021).

Virtual communities provide new platforms for consumers to share knowledge and information about brands and products, and their popularity is skyrocketing due to the great improvements made in mobile internet technology and smartphones in recent years, unleashing the diversity of global consumer demand and supplier offerings (Chang et al., 2009). As sources of knowledge, members in virtual communities play a significant role in consumer collecting action, as affirmed in related research on virtual community functions in marketing (Hsu et al., 2011). The use of the brand community to create contact points, attract and enhance the relationship between consumers and brands (Lin et al., 2020), to a certain extent, to promote consumer participation, thereby attracting more customers. Participation in virtual communities can promote consumer interaction in knowledge and information sharing (Zhou and Lu, 2008), stimulate sustainable involvement, and foster a sense of brand identity and loyalty. Normally, the participants in traditional social activities are already familiar with each other, and the absence of this context in virtual communities is beneficial for the breakage of regional limits, mutual communication between members of common interests, and the enlargement of exchange scope (Wang, 2010; Liang et al., 2021).

With the emergence of the relationship marketing era, companies universally started focusing on the relationship between brands and consumers, hoping to convey the idea of brands to consumers through various means. In marketing practices, consumer loyalty to brands is one of the responsibilities of concern to the brand principal. Brand success is based on long-term cooperation with consumers, and importance must be attached to the construction and maintenance of such cooperation (operationalized in sustained and frequent interactions). Many scholars regard the consumer–brand relationship as an everlasting focused object, with related topics being the exploratory target. According to related research on branding, what consumers bring to brands is beyond the rudimentary interaction between people and objects (i.e., products). Some characteristics of interpersonal relationships exist in branding due to the special emotional affiliations and responses of the consumers (Fournier, 1998). Thus, the relation and communication between consumers and brands must be explored in the context of wider emotional responses to brand offerings.

The process of knowledge sharing includes giving (i.e., sharing) and receiving; correspondingly, members of virtual communities can be divided into sharers and receivers. Sharing and getting knowledge is essentially product involvement behavior that promotes brand loyalty. Studies usually focus on the knowledge sharer as the focus of research, but the reality is that the receiver members are ultimately of more import for branding (and sales), acquiring knowledge that helps them learn more about the brand. Therefore, it is meaningful to study the influence of knowledge sharing on consumer–brand relationships from the perspective of receivers. This study takes knowledge receivers as the research subject, analyzing the impact of knowledge-sharing quality and characteristics on the consumer–brand relationships.

Research on knowledge sharing mainly focuses on antecedent analysis, while few studies have considered the subsequent effects of knowledge sharing. From the perspectives of technology acceptance, psychology, sociology, and system theory, the authors studied causes that directly or indirectly affect knowledge sharing in virtual communities, including community environment, individual characteristics, expectation or motivation, and their subsequent impacts on brand attitude and brand reputation.

The theoretical model of the influence of knowledge sharing on the consumer–brand relationship in virtual communities was constructed with reference to the stimulus–organism–response (SOR) model, and the sense of virtual community was taken as the mediating variable. Using this model, this study undertakes to verify whether product involvement plays a moderating role in the impact of knowledge sharing on the sense of virtual community.

## Literature Review

The SOR model is derived from psychology and explains the effects of environmental characteristics on user behavior and psychological activities, and Mehrabian and Russell (1974) proposed SOR theoretical models based on environmental psychology, S represents the stimulus of the external environment, which affects the subject, O represents the cognitive organism, and after some external environmental stimuli, produces the corresponding psychological activity and reacts accordingly is defined as R, such as acceptance or rejection, adoption, or circumvention.

This model was used in studies of advertising and product consumption over many decades from the mid-twentieth century. With the rapid development of e-commerce, the interests of many scholars were aroused by the effect of virtual shopping environment characteristics on competent consumers due to the change of shopping pattern, from substantial/ traditional to online formats. Elements, such as information quality, are considered as stimuli in the internet context, which affect perceptions of consumers in online environments, shaping their attitudes and trust in messages, products, and brands, and thus affecting consumer decisions (Chen, 2007; Hsu et al., 2012). Consumer engagement, perception, and internet involvement are also affected by online information interaction, individual preference, and brand popularity (Huang, 2012). Yu and Xu (2017) exploited a broadcasting platform as a virtual surrounding carrier and defined engagement in terms of the barrage of emotional display within the SOR model, testing the positive effect of engagement experience on the participation of consumers. Xu et al. (2017) constructed the social network site model and studied its impact on large-scale consumer drain. Zhou and Chen (2018) exemplified the positive effect of online payment and service quality on the sense of virtual community and purchaser application. Kaur et al. (2020) surveyed 602 Facebook users to confirm the positive impact of consumer brand engagement (CBE) on brand loyalty. A survey of brand favorites on Facebook showed that self-branding relationships were the driving force for CBE, which was a key factor in inducing brand loyalty (Helme-Guizon and Magnoni, 2019). Cheung et al. (2020) collected data from 187 experienced users of Brazilian social media, and the results showed that consumer–brand participation has an impact on the intentions of consumers to co-create brand value and bought back.

Brand relationship quality (BRQ) (Fournier, 1998) has been an influential model in SOR research, used by Aaker and Brasel (2004) to further show that in the BRQ scale, the correlation between “love and passion” and “interdependence” is too high, and “brand partner quality” is used to describe the limited relationship between consumers and brands, which is also recognized by Xie and Peng (2009). Based on this, Aaker proposed the concept of consumer–brand relationship strength, composed of satisfaction, commitment, intimacy, and self-connection dimensions, which describes the durability and effectiveness of the consumer–brand relationship (Aaker and Brasel, 2004). This research studies the effect of knowledge sharing on the effectiveness of consumer–brand relationships through the sense of virtual community, building the consumer–brand relationship strength model.

## Research Hypotheses

In the traditional community, the cohesion, organizational structure, and communication behavior of the collective are considered by psychologists to form a “sense of community” (Wang, 2010). McMillan and Chavis (1986) defined a sense of community as a perception of belongings produced when the needs of the members are satisfied, or their common beliefs developed. The virtual community, facilitated by internet technology, is an informal community of individuals sharing certain overlapping (though not identical) common interests, and it does not own actual spaces in the same way as traditional communities. Virtual communities are manifest in the interactions of their members through the internet, with the “community” dimension being a cognitive construct among members (Chen et al., 2013). Blanchard and Markus (2004) proved the existence of a “sense of community” in virtual communities by using a journalism community as the research object. In general, the sense of virtual community has been the focus of many scholars for two reasons: it is one extended concept of sense of community, which is relatively complex and novel; and it strengthens the vitality of the virtual community and provides a theoretical framework for virtual community trends, whose investigation is of immense importance to numerous fields, not least in e-commerce.

The virtual sense of community still lacks a uniform definition. It is generally accepted to explain and study the sense of virtual community in terms of sense of membership, influence, and sense of immersion. *Sense of membership* indicates the identity and belongingness of members, while *influence* refers to the influence of a member on the community, and *sense of immersion* indicates the degree to which members are engaged and embedded (e.g., willing to spend time in) the community. This study selects two dimensions: the sense of membership and sense of immersion, because we take the receivers of knowledge sharing as the research subject, regardless of its impact on other members of the community.

Although there is no relative research on the effect of knowledge sharing on the sense of virtual community, some scholars do study the quality of knowledge sharing, the professional capability of knowledge sharing, and the effect of community status on perception on utility and satisfaction (Petty and Cacioppo, 1986; Cheung et al., 2008; Yang, 2013). Demand meeting is the premise of a sense of virtual community (Koh and Kim, 2003; Ellonen, 2007). The quality of information and the reliability of information sources affect the degree of acceptance through perceiving the utility, authority, and reliability of information to satisfy the information collection needs of consumers (Liang and Yang, 2016). Kim and Koh pointed out that the sense of virtual community manifests the state of mind of the consumers, satisfaction with participation in good experiences, and value achieving. Ellonen (2007) advocates that satisfaction is a driving force in the sense of virtual community. Based on the above analysis, the following research hypothesis is proposed:

**H1:** The quality of knowledge sharing positively affects the sense of virtual community.
**H1a:** The quality of knowledge sharing positively affects the sense of membership.
**H1b:** The quality of knowledge sharing positively affects the sense of immersion.

Chaiken and Trope (1999) argued that the perceived authenticity, credibility, and expertise of the sender are fundamental elements in judging whether the information is reliable or not in knowledge sharing. The reliability of traditional information sources relies on profession, experience, and credibility; the more professional the information is, the more reliable the information conveyed by supporters will be perceived to be (Martin and Lueg, 2013). Thus, the professional capability of knowledge sharers in virtual communities will affect consumer perceptions of information utility. Chang et al. (2011) demonstrated that the professional capability of information senders and their social status affect risk perception of the virtual community members, while Moon and Kim (2001) discovered that valuable information could effectively produce a sense of belonging and enhance loyalty. Based on the above analysis, the following research hypothesis is proposed:

**H2:** Professional capability positively affects the sense of virtual community.
**H2a:** Professional capability positively affects the sense of membership.
**H2b:** Professional capability positively affects the sense of immersion.

Tonteri (2011) pointed out that participation of consumers in virtual communities has a purpose, and the formation and enhancement of virtual community sense of users are based on meeting expectations. Yang (2013) researched the community status of knowledge-sharing senders in virtual communities and empirically proved the influence of community status characteristics of knowledge-sharing senders on the perceived information of users. Knowledge sharers in virtual communities enhance and consolidate their status in the community by sharing high-quality and accurate knowledge. To get continuous attention from users, the shared content must meet their actual needs. Based on the above analysis, the following research hypothesis is proposed:

**H3:** Community status positively affects the sense of virtual community.
**H3a:** Community status positively affects the sense of membership.
**H3b:** Community status positively affects community immersion.

The strength and depth of the relationship between consumers and brands determine brand equity (Fournier, 1994; Chu and Chan, 2009) pointed out that through the virtual brand community, enterprises can identify consumers who are interested in the brand, to obtain feedback and potential needs of loyal consumers, which is helpful to maintain consumer relationships. McMillan and Chavis (1986) proved that the virtual brand community can help virtual community members to form a sense of “weness,” which is promoted by the awareness of “we” to form a commitment; the stronger the virtual community of members, the stronger the sense of commitment to the brand (Liu and Yang, 2012). Commitment refers to the relatively stable relationship between consumers and brands (Ma and Wang, 2015).

Wu and Wang (2015) proposed that virtual community identity can be divided into identity, community identity, and brand identity between members, gathered in virtual brand communities, having the same preferences of consumers. Communities based on common interests are amenable to resonance with the associated identity and brand identity, in which the community itself has a promoting effect. Identity can also be promoted by members *via* community identity aligned with brand recognition. The sense of membership helps users establish a sense of identity and make an emotional commitment to their communities. The emotional dependence of members on the group affects their self-brand connection (Du et al., 2009). Based on the above analysis, the following research hypothesis is proposed:

**H4:** Sense of membership positively affects the consumer–brand relationship.

The sense of immersion is associated with investing extraordinary time and energy in a community (e.g., beyond what would be expected of individual conventional web browsing). Achieving a sense of immersion entails costs for users, who are more likely to participate in the virtual community to generate positive emotions, which reinforces the commitment to the virtual community. DeLone and McLean (1992) advocated that the feeling of the members in the virtual environment in the process of interaction determines the emotional commitment to the virtual community. Armstrong and Hagel (1996) argued that the convenience of online communication means that virtual brand communities can be used as a potential way to improve consumer loyalty. Liu and Yang (2010), whose research is from the perspective of identity, pointed out that virtual brand communities can easily cultivate brand identity through the identity of members, and both of them have a positive impact on the brand loyalty of consumers. Zhu et al. (2014) advocated that consumers who participate in the virtual community are more likely to be loyal to the brand, and the sense of virtual community positively affects the loyalty of community members. In the virtual brand community, the brand is the core element, and the immersion of members deepens the feelings of attachment of the virtual brand community and the relationship between members. Based on the above analysis, the following research hypothesis is proposed:

**H5:** Sense of immersion positively affects the consumer–brand relationship.

From the above analysis, it can be seen that in the post-effect research on knowledge sharing in virtual communities, the influence effects are all exerted by the integration of virtual communities and internal relationship mechanism; knowledge sharing in virtual communities influences brand attitude and brand reputation through the integration of brand communities or internal relationship. Some scholars studied the impact of enterprise public information, consumer interaction, user participation, online experience, and other variables on the consumer–brand relationship. In the virtual community, consumer knowledge sharing involves the public information of enterprise brands, consumer experience information, interaction, and question answering. In addition, from the perspective of identity, scholars put forward the logic of community identity deepening brand recognition, whereby virtual community users enhance their brand recognition through community recognition.

Huang et al. (2015) pointed that when consumers acquire or share content, information and good experiences can meet their needs through community identity, which affects consumer loyalty, and community identity plays an intermediary role in this. The mediating role of virtual community identity has also been verified in the influence of knowledge sharing on the brand attitude of consumers (Sun, 2018). Consumer interaction to learn more about products and brands and the increase in community identity also enhanced the commitment of the customer to the community and feelings, so that they were more willing to participate in community brand building, which would enhance the brand commitment of the customer (Wei and Li, 2021). Based on the theory of social capital, Xu et al. (2016) studied the influence of virtual communities on organizational citizenship behavior and introduced the sense of virtual community as an intermediary variable. They empirically proved that social capital in virtual communities can influence organizational citizenship behavior through the sense of virtual community, and they proved the mediating role of virtual community sense. Li (2018) investigated the experience of loyalty in mobile fitness communities, empirically demonstrating that community identity plays a mediating role in experience and loyalty. There are two basic conditions for an individual to form social identity: to have a specific group identity and to be aware of the value and emotional significance of this identity. Because the knowledge sharing of virtual brand communities can enable consumers to know more about brand products, this deepens brand understanding and the sense of the significance of participating in the community and establishes and reinforces the relationship with the brand. Based on the above analysis, this research hypothesizes that:

**H6:** Sense of virtual community mediates the influence of knowledge sharing on consumer–brand relations.
**H6a:** Sense of virtual community mediates the influence of knowledge-sharing quality on consumer–brand relationships.
**H6b:** Sense of virtual community mediates the influence of professional capability on consumer–brand relations.
**H6c:** Sense of virtual community mediates the influence of community status on consumer–brand relationships.

Product involvement refers to the degree of correlation between the product attributes perceived by individuals and their needs, values, and interests, which affects the degree of effort to purchase (Zaichkowsky, 1985). Low consumer product involvement entails that consumers do not spend time collecting information when purchasing but will look for the most labor-saving and concise method and will also rely more on subjective cognition to make decisions. The higher the degree of consumer product involvement, the more consumers will actively seek for multiple channels to obtain relevant information (Wang et al., 2016), and compare various information schemes, in which sense they will be more likely to listen to “professionals” (Jin, 2007; Li et al., 2015). The virtual brand community provides consumers with a high involvement scene, and official information and high-quality knowledge sharing can increase the sense of trust and recognition of consumers (Yang, 2015). Therefore, if consumers tend to collect more information when they are more involved in consumer products, the virtual brand communities can provide consumers with a platform to acquire knowledge and information, meet the needs of consumers for information search, and can be conducive to the formation of community recognition and immersion. When the degree of involvement of consumer products is low, consumers are more inclined to make decisions quickly and will not immerse themselves in the virtual brand community, so the possibility of identification and immersion in the virtual brand community will be reduced. Therefore, the degree of involvement of consumer products will have different effects on their sense of virtual community. Based on this analysis, the following hypothesis is postulated:

**H7:** Product involvement plays a positive regulatory role in knowledge sharing affecting virtual community sense.
**H7a:** Product involvement plays a positive regulatory role in knowledge-sharing quality affecting the sense of virtual community.
**H7b:** Product involvement plays a positive regulatory role in professional capability affecting the sense of virtual community.
**H7c:** Product involvement plays a positive regulatory role in community status affecting the sense of virtual community

**Figure 1:**
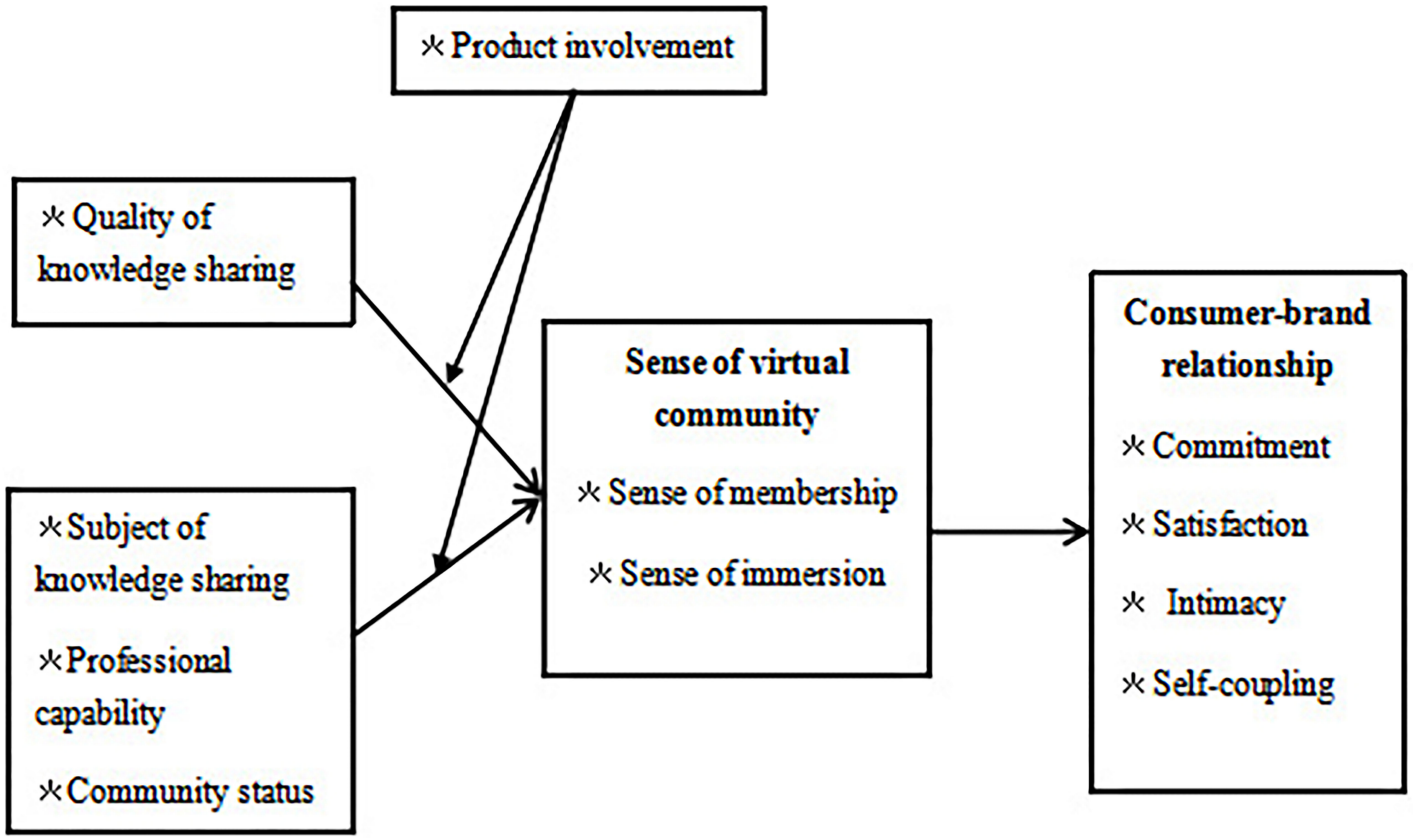
Proposed model.

In conclusion, considering knowledge sharing in virtual communities, the sense of virtual community, and the relationship between consumer–brands, the research hypotheses of this study were developed based on theoretical analysis, and the conceptual model of the influence of knowledge sharing on consumer–brand relationship was constructed by referring to SOR model and information acceptance model, as shown in Figure 1.

## Research Methodology

The research is based on an individual analysis. The design of the research is cross-sectional. First, a small sample of pre-research, through *WeChat (popular social software in China)* sharing a total of 83 questionnaires collected SPSS 22.0 analysis to obtain good confidence of the questionnaire, and then through the “questionnaire star” formally collected questionnaires. Pre-research is conducted to ensure the quality of the questionnaire and the validity of the final data (Luo, 2021).

According to the 44th statistical report on the development of China's Internet issued by China Internet Network Information Center (CNNIC), the 10–39-year-old Internet users account for 65.1% of the total Internet users, of which the 20–29-year-old account for the highest proportion, reaching 24.6%. In addition, among the netizens in China, students are the most, accounting for 26.0%. The theme of this study is a virtual community, and the respondents should be the groups with virtual community participation experience. As the main force of network participation, college students have a higher education level and strong stickiness to the network, so this study chooses college students as the main survey objects. The questionnaire is mainly aimed at the college students of Henan Province. The author, with the convenience of the profession of teachers, gives questionnaires to the students in Henan University, Zhengzhou University, Henan University of Technology, and other universities. The teachers help them understand the problems in the e-commerce experimental course and guide them to answer them carefully.

### Measures

All the variable scales were sourced from previous literature, modified according to the particular study context: knowledge sharing, with three dimensions (quality of knowledge sharing, professional capability, and community status). The scale of DeLone and McLean (1992) is used to measure the quality of knowledge sharing, including four items. It uses the measurement of professional capability of Bansal and Voyer (2000), including four items (Bansal and Voyer, 2000). Community status is measured based on the study of knowledge-sharing subjects in virtual communities by Chang et al. (2011), including three items. Sense of virtual community, with two dimensions (sense of membership and sense of immersion), having four items each (Koh and Kim, 2003). Product involvement is measured based on the research of Zaichkowsky (1985) and Chang et al. (2011), including four items. Consumer–brand relationship, with four dimensions (commitment, intimacy, satisfaction, and self-connection), commitment, intimacy, and self-connection have four items each, and satisfaction has three items (Aaker and Brasel, 2004), all the items are shown in Table 1.

**Table 1:** Sample basic information statistics.

**Sample feature**	**Number (percentage)**
**Gender**	
Male	182 (50.4)
Female	179 (49.6)
**Age (years)**	
<18	1 (0.3)
18–25	91 (25.2)
26–30	124 (34.3)
31–40	120 (33.2)
>40	25 (6.9)
**Education**	
Junior high and below	2 (0.6)
Senior high school	16 (4.4)
College	306 (84.4)
Master's degree +	37 (10.2)
**Monthly income (yuan)**	
<300	68 (18.8)
3,001–5,000	85 (23.5)
5,001–8,000	132 (36.6)
>8,000	76 (21.1)
**Product category**	
Cosmetics	120 (33.2)
Mobile phone	162 (44.9)
Motor vehicles	40 (11.1)
Other category	39 (10.8)
**Role in community**	
Knowledge-sharing	49 (13.6)
Information-acquisition	273 (75.6)
Chatting	20 (5.5)
Other categories	19 (5.3)
**Years of using community**	
<1	129 (35.7)
2–3	149 (41.3)
3–5	62 (17.2)
>5	21 (5.8)

### Data Collection and Sample Characteristics

Data collection were done through the questionnaire distribution platform provided by “questionnaire star” online, 361 valid questionnaires were eventually used. The data were analyzed using SPSS 22.0 and AMOS 24.0 (Zhang et al., 2019; Kim et al., 2021).

A pre-test was carried out whereby 83 valid questionnaires were collected through WeChat, and SPSS22.0 was used to test reliability and validity test. The results showed that the Cronbach's alpha coefficient of all variables was >0.8, and the Kaiser-Meyer-Olkin (KMO) coefficient of all variables was >0.8, which proved that the questionnaire had good reliability and validity. Subsequently, through the questionnaire distribution platform provided by the network company “questionnaire star,” the electronic questionnaire is formed, and then people who have participated in the virtual community experience are selected in the circle of friends to forward the network link of the electronic questionnaire, and they are entrusted to select the appropriate subjects in their circle of friends to forward the questionnaire. The author gives them a serious account of the matters related to the questionnaire survey. Finally, 361 valid questionnaires of the 401 collected responses were reserved through the careful inspection and elimination of the unqualified questionnaires (e.g., questionnaires with obvious similarity and regularity), with a final valid response rate of 90%. The basic information on the sample is shown in Table 1.

The theme of this study is a virtual community, and the respondents should be the groups with virtual community participation experience. As the main force of network participation, college students have a higher education level and strong stickiness to the network, so this study chooses college students as the main survey objects. The questionnaire is mainly aimed at the college students of Henan Province. The author, with the convenience of the profession of teachers, gives questionnaires to the students in Henan University, Zhengzhou University, Henan University of Technology, and other universities. The teachers help them understand the problems in the e-commerce experimental course and guide them to answer them carefully.

According to the data, the proportion of men and women in the survey sample was almost equal, accounting for 50.4 and 49.6% of the total effective sample, respectively. Most were aged between 18 and 40, and the distribution was relatively average, which is consistent with the actual situation of the distribution of groups participating in the virtual community. The majority (84.4%) had a college education. Monthly income distribution was relatively equal (<3,000 yuan: 18.8%, 5,001–8,000 yuan: 36.6%). The types of virtual brand communities that the survey sample focuses on are mainly cosmetics and mobile phones, accounting for 33.2 and 44.9% of the total sample, respectively. Among them, up to 75.6% of people participate in virtual brand communities for the purpose of obtaining information. This sample is appropriate to achieve the study aim of exploring the influence of knowledge sharing in virtual communities on consumer–brand relationships from the perspective of knowledge-sharing receivers.

### Measurement Model Analysis

Reliability refers to the degree of consistency of results obtained when repeated measurements of the same object are repeated using the same method. Cronbach's alpha confidence coefficient is the most commonly used confidence coefficient, applicable to attitude, opinion questionnaire (scale) confidence analysis, the confidence coefficient of the scale is best above 0.7 (Zhang, 2017). The specific values of each variable in this study are shown in Table 2, the Cronbach's alpha values of all variables were >0.7 indicating that the internal consistency of the scale was good.

**Table 2:** Variable reliability testing and confirmatory factor analysis.

**variable**		**Factor loading**	**Cronb-ach's α**	**AVE**	**CR**
Quality of knowledge sharing (QKS)	Knowledge sharing is relevant to the brand	0.698	0.803	0.508	0.805
	Knowledge sharing is accurate	0.746			
	Knowledge sharing is complete	0.663			
	Knowledge sharing is reliable	0.743			
Professional capability (PC)	The sender is familiar with the brand	0.703	0.727	0.471	0.727
	The sender is an expert	0.654			
	The sender has extensive experience in purchasing or using	0.702			
Community status (CS)	The sender posts lots of quality information	0.638	0.706	0.448	0.708
	The sender is prestige	0.723			
	The sender posts and replies positively	0.645			
Sense of membership (SM)	I feel like a member of this community	0.747	0.865	0.616	0.865
	The members are like my friends	0.793			
	I like the members of the community	0.803			
	I belong in this community	0.797			
Sense of immersive (SI)	I spent lots of time in the community	0.838	0.812	0.539	0.820
	I log in the community often	0.799			
	I spent more time than expected	0.736			
	My involvement has affected other arrangement	0.525			
Product involvement (PI)	The brand is important to me	0.825	0.859	0.606	0.850
	The brand is relevant to me	0.736			
	The brand is meaningful to me	0.759			
	The brand is useful to me	0.791			
Commit-ment (COM)	I am loyal to the brand	0.788	0.816	0.553	0.820
	I am willing to wait when the brand unavailable	0.689			
	The brand is my first choice	0.748			
	I will support the brand when it is in trouble	0.692			
Intimacy (INT)	The brand meets my needs	0.690	0.798	0.500	0.800
	I would like to recommend the brand to others	0.677			
	I am familiar with the brand	0.741			
	I know about the brand	0.721			
Satisfaction (SAT)	I am satisfied with the brand	0.706	0.734	0.480	0.735
	I am glad with the brand	0.699			
	The brand exceeded my expectation	0.675			
Self coupling (SC)	The brand reflects my personality	0.718	0.822	0.528	0.816
	The brand suits me	0.766			
	The brand express what I want to be	0.658			
	I feel connection to the brand	0.760			

The scales used in this study are mature scales widely used, and the validity of the scales can be tested and the verification factor analysis can be carried out. The verification factor analysis measures three parts, namely, convergence validity, differentiation validity, and structural validity (Suhr, 2006).

Convergent validity refers to the problem item that measures the same potential traits that fall on the same sub-variable structure and is highly relevant to the measurement measured between the questions. Convergence validity criteria: (1) factor loads should be >0.5, (2) the combined reliability (CR) is >0.7, and (3) the average extraction variance (AVE) should be >0.5. The specific values of each variable in this study are shown in Table 2. The factor loading of each variable was >0.5. The CR values of all variables were >0.7. The average extraction variance (AVE) values of professional capability, community status, and satisfaction were 0.471, 0.448, and 0.480 > 0.4. AVE values for other variables were >0.5, so the convergent validity of the measurement scale was good.

Discriminant validity represents the degree of correlation and difference between potential variables, which can be distinguished from other sub-variables when the square root of the AVE of the sub-variable should be larger than the correlation coefficient between itself and the other potential variables (Suhr, 2006). The correlation coefficient and discriminant validity of each variable are shown in Table 3. The square root of AVE for each variable is larger than its correlation coefficient with other latent variables, indicating that the discriminant validity of the scale was good, and the discriminant validity of the scale of each variable in this study reaches an acceptable level. According to the correlation coefficient, knowledge-sharing quality, professional ability, and community status have significant positive correlations with the factors of virtual community sense and consumer–brand relationship, and the positive correlation between the last two is significant. The correlation analysis results offer preliminary support for the associated hypothesis.

**Table 3:** Correlation coefficient and discrimination validity.

	**QKS**	**PC**	**CS**	**SM**	**SI**	**PI**	**COM**	**INT**	**SAT**	**SC**
QKS	0.713									
PC	0.652**	0.686								
CS	0.626**	0.706**	0.669							
SM	0.603**	0.544**	0.590**	0.785						
SI	0.474**	0.405**	0.438**	0.596**	0.732					
PI	0.635**	0.610**	0.628**	0.741**	0.593**	0.778				
COM	0.551**	0.564**	0.584**	0.677**	0.551**	0.729**	0.743			
INT	0.571**	0.626**	0.592**	0.662**	0.517**	0.718**	0.756**	0.707		
SAT	0.593**	0.610**	0.580**	0.605**	0.533**	0.677**	0.739**	0.769**	0.693	
SC	0.578**	0.538**	0.580**	0.642**	0.602**	0.713**	0.732**	0.716**	0.743**	0.726

** *Indicates a significant correlation at the level of 0.01 (bilateral)*.

The results of structural validity are mainly determined by the absolute fitting indicators and value-added fitting indicators of the structural equations, and the fitting indices include *x*^2^/df, Root Mean Square Error of Approximation (RMSEA), Goodness of Fit Index (GFI), Incremental Fit Index (IFI), Tuckre-Lewis Index (TLI), and Comparative Fit Index (CFI). When *x*^2^/df is between 1 and 2, RMSEA < 0.08, and the values of GFI, IFI, CFI, TLI > 0.9, the structural validity is good (Suhr, 2006). The result of the structural validity test based on AMOS24.0: the *X*^2^/df value was 1.766 <2, the RMSEA value was 0.046 < 0.08, the GFI value was 0.859 > 0.8 (close to 0.9); and FIIFI, CFI, and TLI values were all >0.9. Therefore, the structural validity of the measurement scale was good.

In summary, the questionnaire of this study has fine confidence and validity.

### Structural Model Testing

**Figure 2:**
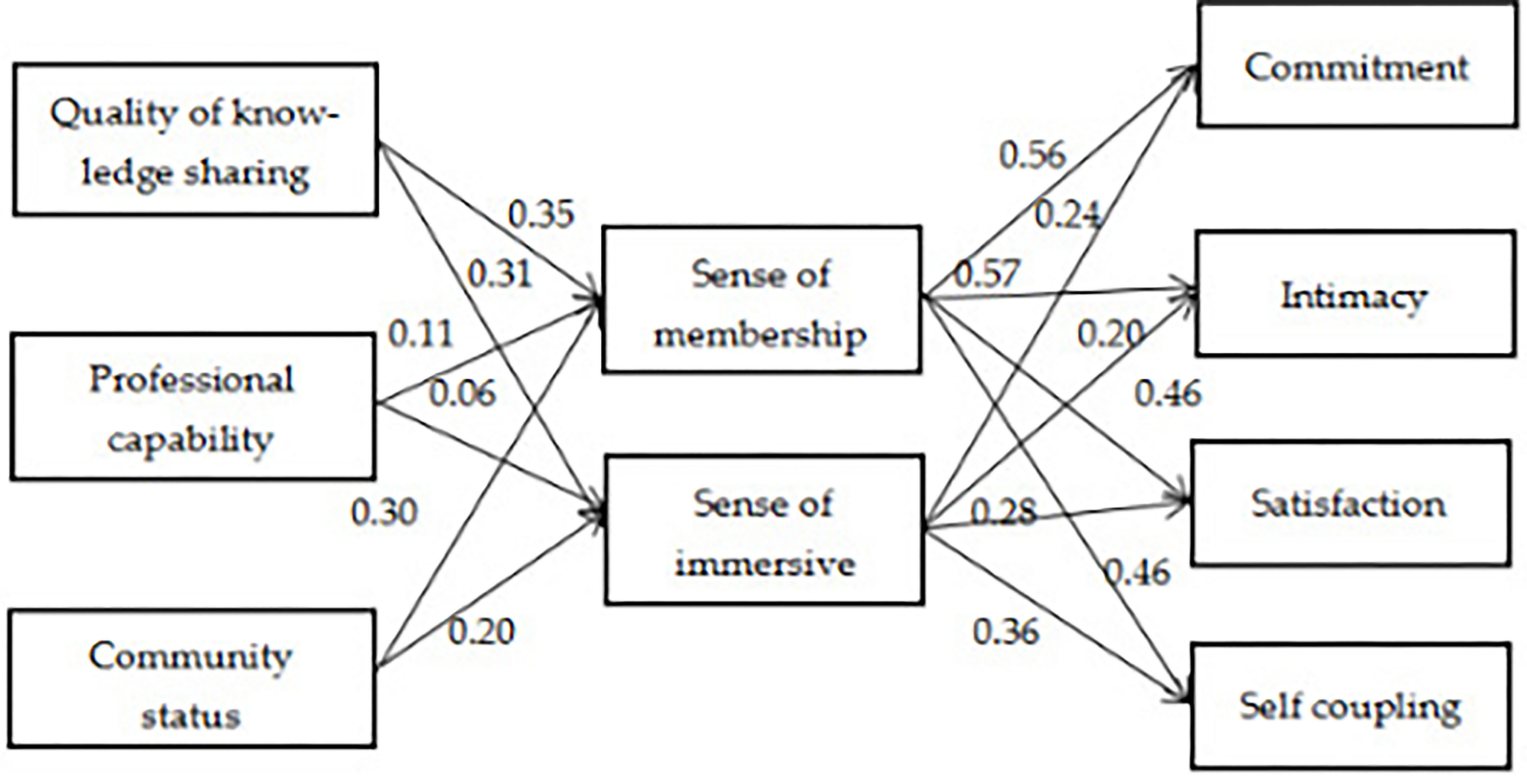
Structural model.

AMOS 24.0 software using the maximum likelihood estimation method was used for Structural equation modeling (SEM). The results shown in Figure 2 indicate that the standardized coefficients are positive, with significant variance indicated by a critical ratio (CR) value of 13.416, not violating estimates (offending estimate).

Table 4 shows the SEM results for the model hypotheses. The CR (Z-value) is the strand error of the path coefficient and SEM: when CR > 3.25 (*p* < 0.001) and CR > 1.96 (*p* < 0.05), the hypothesis is established. The quality of knowledge sharing has a significant positive impact on the sense of immersion and virtual community, with CR values of 6.359 and 4.874, respectively (*p* < 0.001), thus H1 (a and b) is supported. The effect of professional ability (expertise) on the virtual sense of community (membership and immersion) is not significant, with CR values of 1.813 and 0.911, respectively, indicating that H2 (a and b) is not supported. The sense of community status has a significant positive influence on the sense of membership and sense of immersion, with CR values of 5.080 and 2.991, respectively (*p* < 0.001 and *p* < 0.05) supporting H3 (a and b). CR values indicate that sense of membership significantly affects commitment (11.541), intimacy (11.415), satisfaction (8.855), and self-coupling (9.326; *p* < 0.001), supporting H4 (a–d). CR values support the influence of sense of immersion on consumer–brand relationship in terms of commitment (4.909), intimacy, (3.922), satisfaction, (5.319), and self-coupling (7.244; *p* < 0.001), supporting H5 (a–d).

**Table 4:** Significance test of path coefficient.

**Path coefficient**	**S.E**.	**C.R**.	***P***	**Inspection results**	
**H1a:** Sense of membership ← Quality of knowledge-sharing					
0.438	0.0690	6.359	***	Yes	
**H1b:** Sense of immersion ← Quality of knowledge-sharing					
0.392	0.0800	4.874	***	Yes	
**H2a:** Sense of membership ← Professional ability					
0.130	0.0720	1.813	0.070	No	
**H2b:** Sense of immersion ← Professional ability					
0.076	0.0840	0.911	0.362	No	
**H3a:** Sense of membership ← Community status					
0.363	0.0720	5.080	***	Yes	
**H3b:** Sense of immersion ← Community status					
0.250	0.0830	2.991	**	Yes	
**H4a:** Commitment ← Sense of membership					
0.489	0.0420	11.541	***	Yes	
**H4b:** Intimacy ← Sense of membership					
0.458	0.0400	11.415	***	Yes	
**H4c:** Satisfaction ← Sense of membership					
0.365	0.0410	8.855	***	Yes	
**H4d:** Self-coupling ← Sense of membership					
0.412	0.0440	9.326	***	Yes	
**H5a:** Commitment ← Sense of immersion					
0.206	0.0420	4.909	***	Yes	
**H5b:** Intimacy ← Sense of immersion					
0.156	0.0400	3.922	***	Yes	
**H5c:** Satisfaction ← Sense of immersion					
0.217	0.0410	5.319	***	Yes	
**H5d:** Self-coupling ← Sense of immersion					
0.316	0.0440	7.244	***	Yes	
**Assumptions**	**Path coefficient**	**S.E**.	**C.R**.	**P**	**Inspection results**
H1a Sense of membership ← Quality of knowledge-sharing	0.438	0.0690	6.359	***	Yes
H1b Sense of immersion ← Quality of knowledge-sharing	0.392	0.0800	4.874	***	Yes
H2a Sense of membership ← Professional ability	0.130	0.0720	1.813	0.070	No
H2b Sense of immersion ← Professional ability	0.076	0.0840	0.911	0.362	No
H3a Sense of membership ← Community status	0.363	0.0720	5.080	***	Yes
H3b Sense of immersion ← Community status	0.250	0.0830	2.991	**	Yes
H4a Commitment ← Sense of membership	0.489	0.0420	11.541	***	Yes
H4b Intimacy ← Sense of membership	0.458	0.0400	11.415	***	Yes
H4c Satisfaction ← Sense of membership	0.365	0.0410	8.855	***	Yes
H4d Self-coupling← Sense of membership	0.412	0.0440	9.326	***	Yes
H5a Commitment ← Sense of immersion	0.206	0.0420	4.909	***	Yes
H5b Intimacy ← Sense of immersion	0.156	0.0400	3.922	***	Yes
H5c Satisfaction ← Sense of immersion	0.217	0.0410	5.319	***	Yes
H5d Self-coupling ← Sense of immersion	0.316	0.0440	7.244	***	Yes

^***^p < 0.001,

^**^p < 0.01. C.R. value is Z-value.

### Mediating Effect Test

The mediating effect is tested by the Bootstrap method.

The SPSS macro model by Cao and Xiang (2012) was used to test the mediating role of virtual community sense in the influence of knowledge-sharing quality and consumer–brand relationship. The sense of virtual community in this study is divided into two dimensions: the sense of membership and sense of immersion. The consumer–brand relationship is divided into four dimensions: commitment, intimacy, satisfaction, and self-coupling. The results shown in Table 5 indicate that knowledge-sharing quality has a significant effect on commitment (*t* = 12.32, *p* < 0.01), sense of membership perception has a significant effect on commitment (*t* = 13.91, *p* < 0.01), and when the mediating variable sense of membership perception is added, knowledge-sharing quality still has a significant effect on commitment (*t* = 4.75, *p* < 0.01), and sense of membership perception has a significant effect on commitment (*t* = 11.58, p < 0.01). In addition, the mediating effect of sense of membership did not include 0 at the upper and lower limits of bootstrap 95% CI, indicating that the sense of membership could influence commitment appropriately through knowledge sharing. This mediating effect (0.37) accounted for 59.63% of the total effect.

**Table 5:** Mediating effect.

**Mediating effect test**	**Direct effect**			**Indirect effect**			**Mediating effect (%)**
	**Effect**	**95% CI**		**Effect**	**95% CI**		
		**BootLLCI**	**BootULCI**		**BootLLCI**	**BootLCI**	
KQ → SM → COM	0.26	0.15	0.36	0.37	0.29	0.46	59.63
KQ → SM → INT	0.28	0.00	0.18	0.31	0.24	0.38	52.54
KQ → SM → SAT	0.37	0.00	0.26	0.24	0.17	0.30	39.34
KQ → SM → SC	0.36	0.00	0.24	0.32	0.24	0.40	47.06
KQ → SI → COM	0.43	0.00	0.32	0.20	0.13	0.27	31.75
KQ → SI → ZNT	0.44	0.00	0.34	0.15	0.10	0.21	25.42
KQ → SI → SAT	0.45	0.00	0.36	0.16	0.10	0.21	26.23
KQ → SI → SC	0.44	0.00	0.34	0.23	0.16	0.30	34.33
CS → SM → COM	0.32	0.00	0.22	0.33	0.26	0.41	50.77
CS → SM → INT	0.31	0.00	0.22	0.28	0.22	0.35	47.46
CS → SM → SAT	0.34	0.00	0.25	0.23	0.17	0.30	40.35
CS → SM → SC	0.36	0.00	0.25	0.30	0.22	0.39	45.45
CS → SI → COM	0.47	0.00	0.55	0.18	0.11	0.24	27.69
CS → SI → ZNT	0.46	0.00	0.37	0.14	0.08	0.19	23.33
CS → SI → SAT	0.43	0.00	0.34	0.15	0.10	0.20	25.86
CS → SI → SC	0.45	0.00	0.35	0.21	0.15	0.28	31.82

The results showed that the mediating effects of sense of membership on knowledge-sharing quality, intimacy, satisfaction, and self-coupling, and the judgment method were 0.31, 0.24, and 0.32, respectively, accounting for 52.54, 39.34, and 47.06% of the total effects.

Through the analysis, it was concluded that the mediating effects of the sense of virtual community on immersive community status in terms of commitment, intimacy, satisfaction, and self-coupling were as follows: accounting for the related percentages of the total effect: commitment (0.33, 50.77%), intimacy (0.28, 47.46%), satisfaction (0.23, 40.35%), and self-coupling (0.30, 45.45%). The mediating effects of the sense of virtual community on consumer–brand relationships were as follows: accounting for the related percentages of the total effect: commitment (0.18, 27.69%), intimacy (0.14, 23.33%), satisfaction (0.15, 25.86%), and self-coupling (0.21, 31.82%).

Professional capability has a significant effect on commitment, intimacy, satisfaction, and self-coupling (*t* = 12.93, *p* < 0.01; *t* = 15.19, *p* < 0.01; *t* = 14.59, *p* < 0.01; *t* = 12.93, *p* < 0.01). According to the SEM path analysis results, H4 (a–d) and H5 (a–d) are supported, and H2 (a and b) is not supported. Therefore, H6b needs to be Sobel tested. As shown in Table 6, *p* > 0.05, assuming that the mediating effect of sense of virtual community in professional capability and consumer–brand relationship is not significant.

**Table 6:** Sobel test results.

**Mediating effect test**	**Mediating effect**
PC → SM → COM	The Sobel test is not significant (*P* = 8.568)
PC → SM → INT	The Sobel test is not significant (*P* = 8.067)
PC → SM → SAT	The Sobel test is not significant (*P* = 6.999)
PC → SM → SC	The Sobel test is not significant (*P* = 8.078)
PC → SI → COM	The Sobel test is not significant (*P* = 6.131)
PC → SI → INT	The Sobel test is not significant (*P* = 5.602)
PC → SI → SAT	The Sobel test is not significant (*P* = 5.841)
PC → SI → SC	The Sobel test is not significant (*P* = 6.652)

In conclusion, the mediating variable virtual community sense plays a significant mediating role in independent variables knowledge-sharing quality and community status, and the dependent variable consumer–brand relationship, supporting H6 (a–c). The mediating effect of sense of virtual community in professional capability and consumer–brand relations is not significant; therefore, H6b is not supported.

### Regulation Effect Test

The moderating effect was analyzed through multi-level regression analysis (Table 7). Since the professional ability of structural equation path analysis was not supported for the relationship of sense of virtual community, only the moderating effect of product involvement in the influence of knowledge-sharing quality and community status on the sense of virtual community was analyzed. A total of four models were set up (Models 1–4).

**Table 7:** Results of multilevel regression analysis of effects.

**Variables**	**Sense of membership**		**Immersion**	
	**Model 1**	**Model 2**	**Model 3**	**Model 4**
**Controlling variables**				
Gender	−0.002	−0.010	0.007	−0.004
Age	0.094**	0.101**	0.026	0.032
**Independent variables**				
Quality of knowledge-sharing	0.215***		0.157**	
Community status		0.207***		0.108*
**Adjusted variables**				
Product involvement	0.595***	0.605***	8.965***	0.527***
**Interactive items**				
Quality of knowledge-sharing * Product involvement	0.026		0.069	
Community status * Product involvement		0.041		0.037
*R* ^2^	0.589	0.588	0.373	0.361
*F*	101.602***	101.230***	42.152***	40.110***

*** *p < 0.001*,

** *p < 0.01*,

* *p < 0.05*.

Models 1 and 3 indicated significant effects of product involvement on the quality of knowledge sharing of members and its regulating role in the immersive relationship, the quality of knowledge sharing and product involvement in the interaction of the items in the test model, and the quality of knowledge sharing for members and immersive effect (Model 1 beta = 0.215, *p* < 0.001 and Model 3 beta = 0.157, *p* < 0.01). The quality of knowledge sharing and product involvement degree of the interaction of the members and immersive effect were not significant (Model 1 beta = 0.026, *p* > X and Model 3 beta = 0.069, *p* > 0.05). This indicates that the moderating effect of product involvement on the effect of knowledge-sharing quality on the sense of virtual community is not significant.

Models 2 and 4, respectively inspected product involvement degree of status in the community of members, the regulating role in the immersive relations, community status, and product involvement degree of the interaction of the items in the test model, with significant effects of the community status of members and the influence of the immersive effect (Model 2 beta = 0.207, *p* < 0.001 and Model 4 beta = 0.108, *p* < 0.05), but the position and degree of product involvement in community interaction on members and the immersive effect were not significant (Model 2 beta = 0.041, *p* > X and Model 4 beta = 0.037, *p* > 0.05), which explained that product involvement degree of status in the community of the regulating role in the effect of the virtual community was not significant, thus H7 (a–c) is not verified.

## Discussion and Conclusion

### Discussion

The theoretical significance of this research is to construct a conceptual model, which builds the model among the virtual community, knowledge sharing, and consumer–brand relationship. Using the sense of virtual community as an intermediary variable, discuss the influence paths of knowledge sharing on consumer–brand relationship.

In the previous research on knowledge sharing in virtual communities, the research results focus on the antecedents that affect knowledge sharing (Li, 2020; Zhang, 2020), which explains why consumers or users share content and information, the content user-generated and shared and impact of the information on other users or companies. However, there are few research results on the aftereffects of knowledge sharing in virtual communities. This study puts forward the hypothesis that knowledge sharing affects the strength of consumer–brand relationships through virtual community perception enriches the content of the aftereffects of knowledge sharing and improves the theory of knowledge sharing.

First, the empirical results confirm the positive impact of knowledge sharing on the sense of virtual community. This result is consistent with the previous research (Li and Zhang, 2018). The effects of knowledge sharing in virtual communities are mainly reflected in brand attitudes and brand reputation. The influences on knowledge sharing are generally through the integration of virtual brand communities and internal relationship mechanisms. Users actively participate in community activities since they can obtain benefits. The stronger the trust between members, the greater the perception of interest of consumers are more willing to share information. Besides, the social needs of consumers also promote consumers to participate in the activities of virtual communities.

Second, the empirical results confirm the positive impact of virtual community on the consumer–brand relationship. Previous studies (Huang et al., 2015; Liu et al., 2018) have shown that community recognition of users can promote their brand recognition. The time and energy invested by users in the virtual community will deepen the brand identity, thereby building an intimate and stable relationship with the brand. The consumer–brand relationship has a positive impact on brand development (Sun, 2018). A brand with a loyal customer group has the foundation of brand equity, which is helpful to the improvement of brand equity.

According to the results of the Bootstrap test, the sense of virtual community is a part of the intermediary in the influence of the quality of knowledge sharing and community status on the consumer–brand relationship. Relevant studies have shown that virtual community knowledge sharing affects brand attitudes and brand reputation through brand community integration or internal relationships (Li, 2018; Wei and Li, 2021). The sense of virtual community means the recognition and immersion of the community and emotional expression of the users of the virtual community, high-quality information, high authority shares of the sharers will increase. The recognition and immersion of the users in the community strengthen the connection between consumers and the brand. According to the analysis results of the structural equation model, the influence of the professional ability of the virtual community knowledge sharers on the virtual community is not supported. The Sobel test result shows that the professional competence of the virtual community has no significant mediating effect on the consumer–brand relationship. In addition, Rubio et al. (2020) proposed that sharers with professional knowledge are more confident to participate in knowledge sharing activities, are more willing to use professional knowledge to contribute to the community, and put forward suggestions on brands or services to realize their value. Sharers will get a high level of accomplishment and maintain a high degree of contact with the community and brand. The reason for not reaching a similar conclusion may be that the subject of this study is the receiver of knowledge sharing. Users make their judgments by browsing the shared content, and there will be inconsistencies in the perception of the professional abilities of the sharers. There will also be differences in identity.

Through multi-level regression, the detection of product involvement in the moderating effect of knowledge sharing on the sense of virtual community is not supported. Existing studies have proved that under the condition of high product involvement, the influence of information quality on perceived usefulness is greater than that of low product involvement. Under the condition of low product involvement, the effect of community status on perceived usefulness is more important. It is greater when the degree of involvement is high (Liu et al., 2018). Zhou and Lin (2020) pointed out that the degree of product involvement has a negative moderating effect in the interaction of advertising on consumer perception.

### Conclusions

Based on the reviewed literature, the theoretical model and study hypotheses were proposed. SPSS22.0 and AMOS24.0 were used to analyze the data and verify the hypothesis proposed in this study. The conclusions are as follows.

The impact of professional capability on the sense of membership and sense of immersion is not verified. This is possibly because the professional capability of the sender is high, which reduces the perceived risk of consumers and affects their purchase decision, but the identity and immersion of the virtual community were not significant. H2 was not verified. Knowledge-sharing quality and community status have significant positive effects on the sense of membership and immersion. Hypotheses H1 and H3 were verified.
The sense of virtual community has a significant positive effect on consumer–brand relationships, and the heart of virtual brand community relations is the relationship between the user and the brand (among members of virtual communities). The higher the immersive feeling, the more easily affected users are by the inside of the virtual community, thus they are more likely to build their relationship with the brand, and this relationship is of greater intensity. Hypotheses H4 and H5 were verified.
The results show that the mediating effect of the sense of virtual community is supported, and knowledge sharing in virtual communities has an impact on the consumer–brand relationship through virtual community sense. H6 is verified.
The moderating effect of product involvement between knowledge sharing and virtual sense of community was not verified. The interaction items of knowledge-sharing quality, community status, and product involvement have no significant influence on the sense of virtual community (namely, high or low degree of product involvement in regulating role between knowledge sharing and virtual community). H7 (the product involvement regulation hypothesis) was not verified.

### Management Enlightenment

According to the research in this study, knowledge-sharing quality, professional ability of sender, and community status of the virtual community can positively influence the strength of consumer–brand relationship through the sense of membership and immersion of virtual community, which provides a new idea for enterprises to construct and maintain consumer–brand relationship through knowledge-sharing function.

First, the quality standards of knowledge sharing in virtual communities were defined, encouraging members to share and create brand knowledge. The quality of knowledge sharing affects the sense of virtual community and consumer–brand relationship and improves the real reliability and readability of shared knowledge or data, which requires the realization of illustrated or simple and understandable language. The virtual community platform can refer to the opinions of members to develop the corresponding knowledge-sharing template. In addition, community members (potential consumers) are encouraged to create and pass on detailed and specific brand knowledge, and rewards are given for high-quality information that can have a positive impact on the brand.

Second, the forms of virtual community activities are enriched to guide positive exchanges and interactions, and further enhance the relationship between community members. According to the characteristics of the virtual community, special rights and activities, such as community points, virtual gold COINS rewards, and search and collection, are considered to encourage the senders and receivers of knowledge sharing to actively participate in community topic discussion and knowledge sharing. In addition, offline activities should be actively carried out in the form of product or service experience exchanges to improve actual understanding of the brand of the virtual community consumers, and online sharing should be combined to realize the integration of online and offline, expanding the influence of the enterprise brand, and enhancing the relationship between community members.

Third, a good community environment can be maintained, enhancing a community sense of identity and belonging to increase user viscosity. The virtual community provides consumers with a platform to share and exchange information. Enterprises must pay attention to the maintenance of a good environment for the virtual community, such as classifying the communication sections, providing special communication areas, establishing rewards, identifying mutual fans and other evaluation functions, and enhancing the sense of identity of consumers with the virtual community. At the same time, enterprises should strive to build a unified community goal for consumers in the virtual community, letting consumers realize their value status in the community, and promoting continuous and sustained participation.

### Limitations

This study discusses the mechanism of knowledge sharing on consumer–brand relationships from the perspective of the knowledge-sharing receiver, to provide new ideas for enterprise marketing practice and brand relationship maintenance. The research is not mature and has some limitations. This study mainly collects sample data for mobile phone, cosmetics, and motor vehicle brand communities; it is aimed at commodity communities. However, the pertinence of virtual communities for studies of brand issues is relatively limited, and there were no specific virtual communities for specific kinds of goods in this study, so the implications of the conclusions are relatively weak. Follow-up studies can be conducted on specific virtual communities to explore the mediating role of perceived usefulness in knowledge sharing in virtual communities and perceived belonging to virtual communities.

## Data Availability Statement

The raw data supporting the conclusions of this article will be made available by the authors, without undue reservation.

## Ethics Statement

The studies involving human participants were reviewed and approved by Ethics Committee of Henan University. The patients/participants provided their written informed consent to participate in this study.

## Author Contributions

All authors listed have made a substantial, direct and intellectual contribution to the work, and approved it for publication.

## Funding

The research is supported by Major Program of basic research of Philosophy and Social Sciences in Colleges and Universities of Henan Province (No. 2021-JCZD-02), Cultivation Program for Innovative Team of Philosophy and Social Sciences of Henan University (No. 2019CXTD008), Social Science planning project of Henan Province (No. 2019BJJ017), Program for Science and Technology Innovation Talents at the Universities of Henan Province (No. 2019-cx-012), and Important Project for Educational Science Planning of Henan Province (No. [2020]-JKGHZD-11). The start-up funding for Ph.D. scientific research of Huaibei Normal University of China (No. 03106098).

## Conflict of Interest

The authors declare that the research was conducted in the absence of any commercial or financial relationships that could be construed as a potential conflict of interest.

## Publisher's Note

All claims expressed in this article are solely those of the authors and do not necessarily represent those of their affiliated organizations, or those of the publisher, the editors and the reviewers. Any product that may be evaluated in this article, or claim that may be made by its manufacturer, is not guaranteed or endorsed by the publisher.
